# Prevalence of Adenomas Found on Colonoscopy in Patients With HIV

**DOI:** 10.4021/gr433w

**Published:** 2012-03-20

**Authors:** Ellen Gutkin, Syed A. Hussain, Preeti Mehta, Sang H. Kim, Simcha Pollack, Moshe Rubin

**Affiliations:** a New York Hospital Queens Weill Cornell Medical College, 56-45 Main Street, Flushing, NY, USA; b St. John’s University, 8000 Utopia Parkway, Queens, New York, USA

**Keywords:** HIV, Adenoma, Colonoscopy, Polyp, Colorectal cancer screening

## Abstract

**Background:**

The life expectancy of patients with HIV has increased significantly since the introduction of highly active antiretroviral therapy in 1995. Although this population of patients now carries less risk for the development of AIDS defining illnesses and malignancies, they are still at risk for non-AIDS defining cancers, such as colon, prostate, and breast. Several studies have shown that HIV infected patients have a higher prevalence of advanced colonic neoplasia which occur at a younger age. Our aim is to examine the prevalence of adenomas and adenocarcinoma in HIV patients undergoing colonoscopy.

**Methods:**

HIV patients seen in our gastroenterology clinic and inpatient service undergoing colonoscopy were identified from 2010 - 2011. Indication was screening in 27 patients and diagnostic in 23 patients. Significant lesions were defined as adenomas, serrated polyps, and adenocarcinoma.

**Results:**

Total 50 patients were included in the study, 32 male and 18 female (mean age: 53.6; range 37 - 72 years), 25 patients were African American, 21 were Hispanic, 3 were Caucasian, and one was Indian, 39 patients had undetectable HIV RNA, 30 patients had CD4 lymphocyte counts greater than 500, 20 had CD4 lymphocyte counts less than 500, and 4 patients had CD4 lymphocyte counts less than 200, 52% (26/50) of patients had polyps. Significant lesions (adenomas and serrated polyps) were seen in 34% (17/50) of patients, 39% in diagnostic and 30% in screening procedures (P = 0.56). Males were found to have significant lesions 28% of the time as compared to 44% of females (P = 0.35), 43% of Hispanics had significant lesions versus 24% of African Americans (P = 0.22), 25% of the patients under age 50 were found to have significant lesions, 45% of the patients with detectable HIV RNA levels were found to have significant lesions vs 31% (P = 0.48). Surprisingly, patients with CD4 counts > 500 had significantly more adenomas than those with CD4 counts < 500, 47% vs 15% (P = 0.03). No adenocarcinomas were seen in our patient population.

**Conclusion:**

In our case series of HIV patients the adenoma detection rate was 34% overall, 45% in the patients with detectable HIV RNA levels, and 47% in patients with CD4 counts > 500. Additionally, there was a 25% adenoma detection rate in patients less than fifty years of age. This data reinforces the need for aggressive colon cancer screening in the HIV population.

## Introduction

The life expectancy of patients with HIV has significantly increased since the introduction of highly active antiretroviral therapy in 1995 [1-3]. The number of people living with HIV/AIDS in New York City alone has been estimated to be 109,446 [4]. Seventy six percent of these HIV patients are age 40 or older and 41 percent are age 50 or older [4].

Although this population of patients now carries less risk for the development of AIDS defining illnesses and malignancies, they are still at risk for non-AIDS defining cancers, such as colon, prostate, and breast [1, 2, 3, 5, 6]. Only four cases of colorectal cancer in HIV infected patients were reported, until a series of 12 patients was published by Yeguez and colleagues [1]. Colorectal cancer (CRC) is the third most commonly diagnosed cancer and the third leading cause of cancer death in both men and women in the US, with 103,170 new cases and 51,690 deaths expected in 2012. About 72% of cases arise in the colon and about 28% in the rectum [7]. However, there is a paucity of data on CRC screening and adenoma detection rates in the HIV population [3]. Some studies suggest that HIV infected patients are significantly less likely to be up-to-date or to have ever had a CRC screening test [2].

Several studies have shown that HIV infected patients have a higher prevalence of colonic neoplasms than the general population [1, 8]. Additional studies have found that in these patients tumors develop at an earlier age, are diagnosed in more advanced stages, and have a dismal prognosis [1, 2, 5, 9]. Our aim is to examine the prevalence of adenomas and adenocarcinoma in HIV patients undergoing colonoscopy in the borough of Queens, New York.

## Methods

HIV patients seen in the New York Hospital Queens (Flushing, New York) gastroenterology clinic and inpatient service who were scheduled for colonoscopy were prospectively identified from 2010 to 2011. Data collected on each patient included age, gender, race, and most recent hemoglobin, CD4 lymphocyte count, and HIV RNA level. The indications for colonoscopy, presence of and number of colon polyps, presence of right sided colon lesions, type of polyp, polyp size greater than one centimeter, and presence of any significant lesions were also recorded for each patient. Significant lesions were defined as adenomas, serrated polyps, and adenocarcinoma. The study protocol was reviewed and approved by the Institutional Review Board at our hospital.

All patients received an oral polyethylene glycol-electrolyte solution or a low volume polyethylene glycol solution along with 10 mg of bisacodyl for bowel preparation. The endoscopists who performed the colonoscopies were blinded to the study aims. All retrieved polyps were sent to the NYHQ pathology laboratory for evaluation. The colonoscopy reports and findings were obtained from the EndoSoft (R) database post procedure.

### Statistical analysis

Comparisons of continuous variables between groups were done using the independent t-test with a Satterthwaite correction for unequal variances when required. Fisher’s exact test and chi-squared was used for testing differences among categorical variables. Mean differences are presented with their 95% confidence intervals. P-values less than 0.05 were deemed statistically significant; no multiple-test adjustment to the p-value was done. All analyses were conducted in 2011 using SAS 9.3 (SAS Institute, Inc, Cary, NC).

## Results

Fifty patients were included in the study, 32 male and 18 female (mean (sd) age: 53.6 (8.8); range 37 - 72 years). All patients had a complete colonoscopy from rectum to cecum. Sixteen patients (32%) were younger than age 50. Twenty five patients were African American (50%), twenty one were Hispanic (42%), three were Caucasian (6%), and one Indian (2%). Thirty nine (78%) patients had undetectable HIV RNA levels. In eleven patients (22%) with detectable HIV RNA, the mean viral load was 259,634 (range 62 - 2,531,659 copies/mL). Thirty patients (60%) had CD4 lymphocyte counts greater than 500, twenty (40%) had CD4 counts less than 500, and four patients (8%) had CD4 counts less than 200 (Table 1).

**Table 1:** Patient Demographics and Adenoma Detection Rates (ADR)

Variable:	n (%)	ADR n (%)
Age		P = 0.52
< 50	16 (32%)	4 (25%)
50 - 59	23 (46%)	10 (43%)
60 - 69	9 (18%)	2 (22%)
> 70	2 (4%)	1 (50%)
Sex		P = 0.35
Female	18 (36%)	8 (44%)
Male	32 (64%)	9 (28%)
Race/ethnicity^1^		P = 0.29
Indian	1 (2%)	1 (100%)
White	3 (6%)	1 (33%)
Hispanic	21 (42%)	9 (43%)
African American	25 (50%)	6 (24%)
Undetectable HIV VL		P = 0.48
No	11 (22%)	5 (45%)
Yes	39 (78%)	12 (31%)
CD4		P = 0.03
≤ 500	20 (40%)	3 (15%)
> 500	30 (60%)	14 (47%)
Indication for Procedure		P = 0.56
Screening	27 (54%)	8 (30%)
Diagnostic	23 (38%)	9 (39%)

1 Comparing only African American vs. Hispanic, P = 0.22.

Colonoscopy indication was screening in 27 (54%) and diagnostic in 23 (46%) of patients. The adenoma detection rate was 39% in diagnostic procedures and 30% in screening procedures (P = 0.56). The overall adenoma detection rate was 34% and the range of adenomas per patient was between 1 - 9.

The adenoma detection rate was 28% in men and 44% in women (P = 0.35). In Hispanic patients the adenoma detection rate was 43% and in African Americans it was 24% (P = 0.29). The adenoma detection rate for patients under age 50 was 25%. No relationship between age groups (< 50, 50 - 59, 60 - 69, > 70) and adenoma detection was observed (P = 0.52).

The adenoma detection rate in patients with CD4 lymphocyte counts less than 500 was 15% as compared to 47% in patients with CD4 counts greater than 500 (P = 0.03). The adenoma detection rate in patients with detectable HIV RNA levels versus undetectable HIV RNA was 31% and 45%, respectively (P = 0.48).

Twenty-four percent of patients had right sided lesions. Eight percent of the patients had lesions > 1 cm. Fifty eight percent of patients with adenomas had more than one adenoma at colonoscopy. No adenocarcinomas were seen in our patient population (Table 1).

## Discussion

Several studies have demonstrated that CRC incidence is increasing in the HIV population [3]. The annual incidence of colon cancer among HIV-infected patients was reported to be 0.65 (2 cases per 1000 patient years) from 1989 - 1996 and rose to 2.34 (11 cases per 1000 patient years) from 1997 - 2002 [6]. In the general population, it is estimated that at least 25% of men and 15% of women above age 50 have one or more adenomas found during colonoscopy screening [10, 11]. In 165 HIV patients examined by Bini, the prevalence of neoplastic lesions (adenomas or adenocarcinomas) in the distal colon was found to be significantly higher than in control subjects (25.5% vs 13.1%) [8]. A study by Iqbal found that HIV-infected patients were more likely to have adenomas on screening colonoscopy as compared to non-HIV patients (50 % vs. 23.8%) [8]. Wasserberg concluded that HIV-positive CRC patients tend to be diagnosed at an earlier age (median age 41) and have a more aggressive presentation with less favorable outcome than non HIV patients [9]. In 2009, Bini again reported a significantly higher prevalence of neoplastic lesions in HIV infected subjects than in control subjects (62.5% vs 41.2%) [12].

In concordance with the available literature, our HIV patients had a higher percentage of adenomas detected (34%) at colonoscopy. The adenoma detection rate was even greater in the patients with detectable HIV RNA levels (45%). Surprisingly, even the immunocompetent patients with CD4 counts greater than 500 had a high adenoma detection rate (47%). Of note, there was a high rate of adenomas in HIV patients less than fifty years of age (25%).

Several potential causes of the high rates of non-AIDS defining malignancies have been described in the literature, including: differences in lifestyle and rates of traditional cancer risk factors among HIV patients, such as smoking; immune deficiency and immune activation due to chronic B-cell stimulation and/or cytokine dysregulation; and the oncogenic potential of viral co-infections and HAART [13, 14].

Limitations of our study include small sample size and single center experience. However, we believe it is important to share our data in order to raise awareness of the opportunities for colorectal cancer screening in the HIV population.

Colorectal cancer screening has been underutilized in the HIV patient population [2]. The underuse of CRC screening tests in patients with HIV has important clinical implications and may represent a missed opportunity for cancer prevention [2]. The increased risk of adenomas evidenced by our data further reinforces the need for education and screening targeted specifically towards the HIV population. Colorectal cancer screening for average-risk people begins at age 50 years (or the age of 45 if the patient is African American) [15]. Based on our results and the available literature it may be reasonable to begin screening at age 40 in this high risk population.
